# Application of a Hybrid Model for Predicting the Incidence of Tuberculosis in Hubei, China 

**DOI:** 10.1371/journal.pone.0080969

**Published:** 2013-11-06

**Authors:** Guoliang Zhang, Shuqiong Huang, Qionghong Duan, Wen Shu, Yongchun Hou, Shiyu Zhu, Xiaoping Miao, Shaofa Nie, Sheng Wei, Nan Guo, Hua Shan, Yihua Xu

**Affiliations:** 1 Department of Epidemiology and Biostatistics, School of Public Health, Tongji Medical College, Huazhong University of Science and Technology, Wuhan, China; 2 Hubei Provincial Center for Disease Control and Prevention, Wuhan, China; 3 Tuberculosis Institute for Tuberculosis Control and Prevention of Wuhan City, Wuhan, China; 4 Department of Epidemiology, Johns Hopkins School of Public Health, Baltimore, Maryland, United States of America; 5 Division of Blood Transfusion, Department of Pathology, Johns Hopkins Medical Institutions, Baltimore, Maryland, United States of America; McGill University, Canada

## Abstract

**Background:**

A prediction model for tuberculosis incidence is needed in China which may be used as a decision-supportive tool for planning health interventions and allocating health resources.

**Methods:**

The autoregressive integrated moving average (ARIMA) model was first constructed with the data of tuberculosis report rate in Hubei Province from Jan 2004 to Dec 2011.The data from Jan 2012 to Jun 2012 were used to validate the model. Then the generalized regression neural network (GRNN)-ARIMA combination model was established based on the constructed ARIMA model. Finally, the fitting and prediction accuracy of the two models was evaluated.

**Results:**

A total of 465,960 cases were reported between Jan 2004 and Dec 2011 in Hubei Province. The report rate of tuberculosis was highest in 2005 (119.932 per 100,000 population) and lowest in 2010 (84.724 per 100,000 population). The time series of tuberculosis report rate show a gradual secular decline and a striking seasonal variation. The ARIMA (2, 1, 0) × (0, 1, 1)_12_ model was selected from several plausible ARIMA models. The residual mean square error of the GRNN-ARIMA model and ARIMA model were 0.4467 and 0.6521 in training part, and 0.0958 and 0.1133 in validation part, respectively. The mean absolute error and mean absolute percentage error of the hybrid model were also less than the ARIMA model.

**Discussion and Conclusions:**

The gradual decline in tuberculosis report rate may be attributed to the effect of intensive measures on tuberculosis. The striking seasonal variation may have resulted from several factors. We suppose that a delay in the surveillance system may also have contributed to the variation. According to the fitting and prediction accuracy, the hybrid model outperforms the traditional ARIMA model, which may facilitate the allocation of health resources in China.

## Introduction

China has the second largest burden of tuberculosis (TB) in the world with huge health and economic losses [1]. Meanwhile, the magnitude and pattern of TB may vary with regions because of the diverse population density and uneven economic development in China [2]. An accurate forecast of the TB incidence in each region will contribute to establishing reasonable health projects. Starting in June 2013, the Global Fund will stop funding China for its prevention and control of TB [3]. Thus, effective and efficient allocation of limited health resources becomes more important. Accurate assessment of the TB epidemic situation is also important for conducting early warning preparations and exploring potential influencing factors for an epidemic [4]. Surveillance data are currently collected to evaluate the epidemic situation but without the ability to offer explicit and quantitative direction for the future health plan in China. Therefore, a high-performance mathematical model is needed.

Studying the prediction of incidences of infectious diseases including TB has been an ongoing effort [5–7]. The autoregressive integrated moving average (ARIMA) model is frequently used, based on a linear presumption [8,9]. However, this linear presumption is not always consistent with complex real-world problems which have inherently noisy, non-stationary and chaotic characteristics. Because of the capability of flexible nonlinear modeling, the neural network model has been proposed as a promising supplementation for the ARIMA model [10]. The generalized regression neural network (GRNN) is a class of neural networks with primary advantages of fast learning and convergence to the optimal regression surface [11]. The occurrence and epidemic of tuberculosis is influenced by several factors such as pathogen variation, environmental changes and health interventions. Thus, it is difficult to recognize all the characteristics in the TB incidence series. It has been universally agreed that a combination of different models can increase the chance of capturing various patterns in the series and improve prediction accuracy [12,13]. Therefore, we conducted this study to create a hybrid prediction model composed of the generalized regression neural network and ARIMA model to predict TB incidence. The prediction performance of the new hybrid model was compared with the traditional ARIMA model alone so as to explore the optimized model and provide suggestions for policymakers.

## Materials and Methods

### Study area and data collection

Data of monthly reported TB cases in Hubei Province from Jan 2004 to Jun 2012 were collected from the Hubei Provincial Center for Disease Control and Prevention. Population data were collected from the Hubei Statistics Bureau. Hubei Province has a high tuberculosis incidence, ranking among the top 10 high-incidence provinces in China. As it is one of the most important transportation junctions for the whole country and nearly a quarter of its population are still in poverty, Hubei Province has potential for an outbreak of TB. China has a relatively reliable surveillance and reporting system for TB and the data collection mechanism has been stable since 2004[14,15]. It is mandatory for hospitals to report new TB cases within 24 hours to the local Center for Disease Control and Prevention through the Internet-based disease-reporting system [16]. Because failure to report is now a crime, hospitals take the reporting of TB very seriously. Thus, trends in case notifications can be used as the measure of trends in incidence. 

 To compare the fitting and prediction performances of the two models separately, the data were divided into two parts: Data between Jan 2004 and Dec 2011 were used to construct the models and data between Jan 2012 and Jun 2012 were used to compare the prediction accuracy of the two models. Ethical approval was not necessary because the study was retrospective and the data are for public access. 

### Basic ARIMA model construction

The ARIMA model is often written in shorthand as ARIMA (p, d, q) (P, D, Q) _S_. The orders p, d, q, P, D, Q, _S_ refer to the number of auto-regressive lags, differences, moving-average lags, seasonal auto-regressive lags, seasonal differences and seasonal moving-average lags, and the length of cyclical pattern, respectively. We adopted the Box-Jenkins approach to fit an ARIMA model with three interactive steps of model identification, parameter estimation, and diagnostic checking [17]. Before constructing the model, the time series should be stationary with respect to mean and variance. Several procedures including log transformation, non-seasonal and seasonal difference were applied to stabilize the series. In the stage of model identification, the autocorrelation function (ACF) graph and the partial autocorrelation (PACF) graph were employed to identify the numbers of seasonal and non-seasonal auto-regressive and moving-average terms. The seasonal part of the model should be recognized first and then the non-seasonal part can be figured out from the residual plots. Several plausible models may be taken into account and the optimum model will be selected through model examinations. The parameters of the prediction model were estimated using the maximum likelihood method. Several diagnostic statistics including coefficient of determination (R^2^), mean square error (MSE), Schwarz Bayesian information criterion (SBC), and Akaike information criterion (AIC) were used to evaluate the fitness of the model. Finally, the selected model was applied for predicting the TB incidence in the next six months.

### Construction of the hybrid GRNN-ARIMA model

The generalized regression neural network (GRNN) is a branch of radial basis artificial networks capable of performing kernel regression and other non-parametric functional approximations [13]. The configuration of GRNN consists of four layers: the input layer, pattern layer, summation layer, and output layer. This neural network examines the relationship between each pair of the input vector X and the observed output Y and finally deduces the underlying function [18]. It can be equivalent to the following equation:

$$E\left[Y/X\right]=\int_{-\infty }^{\infty }Yf(X,Y)dY/\int_{-\infty }^{\infty }f(X,Y)dY$$

where X denotes the input series(X1, X2 … Xm), and Y means the predicted values of GRNN. E(Y/X) is the expected value of the output Y with a given input vector X and f(X, Y) is the joint probability density of X and Y [13]. The GRNN does not suffer from the frequently encountered local minima problem compared with other neural networks. The performance of the GRNN depends heavily on the spread factor [19]. A large spread will increase the network’s ability of generalization and decrease the prediction error, but requires a lot of neurons to fit a fast changing function at the same time. 

When constructing the hybrid model, the fitted values of the ARIMA model and the actual values were used as the input and output of the GRNN, respectively. We determined the spread using the method proposed by Specht [11]. Two samples were randomly selected as the testing samples and the other samples were used to fit a network. This fitting network was trained and tested for a range of spread values to determine the optimal spread according to the minimum testing error. Then the short-term predicted values of ARIMA model were used as the input of the constructed GRNN. The network was simulated to revise these values. These revised values were the prediction values created by the hybrid model.

Finally, the fitting and predicting accuracy of the two models was evaluated using the mean square error (MSE), mean absolute error (MAE), and mean absolute percentage error (MAPE). The ARIMA model was created with SAS 9.1; the GRNN-ARIMA model was constructed with Matlab 7.0.

## Results

### Characteristics of the time series of TB report rate

**Figure 1:**
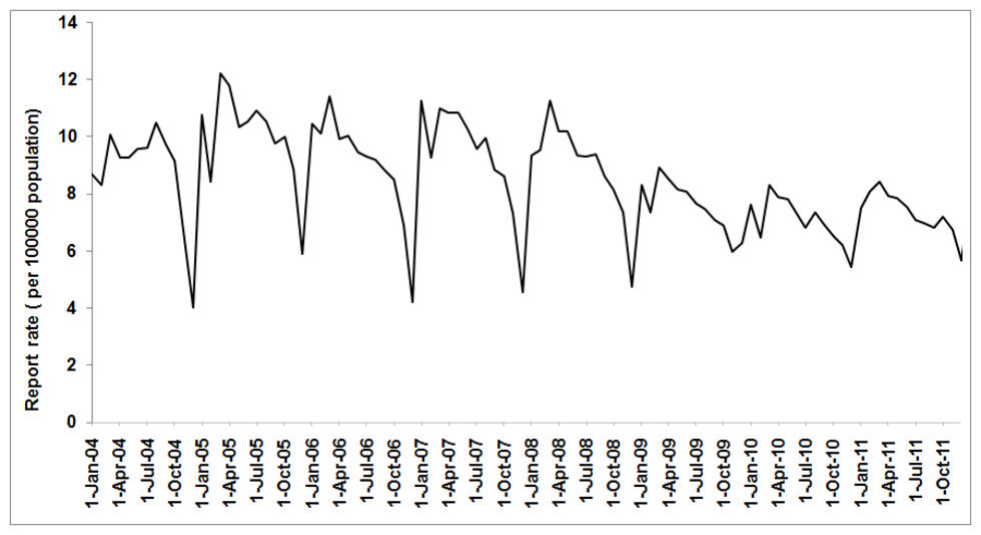
Time series of tuberculosis report rate in Hubei Province, China between Jan 2004 and Dec 2011.

A total of 465,960 cases were reported between Jan 2004 and Dec 2011 in Hubei Province. The report rate of TB was highest in 2005 (119.932 per 100,000 population) and lowest in 2010 (84.724 per 100,000 population). According to the time series of report rate, we found a sharp report rate increase in 2005 and an obviously different fluctuation report rate pattern between 2004 and 2005 (Figure 1). During the study period, an overall decreasing trend in TB report rate was observed. However, there was a slight rise in 2011.

**Figure 2:**
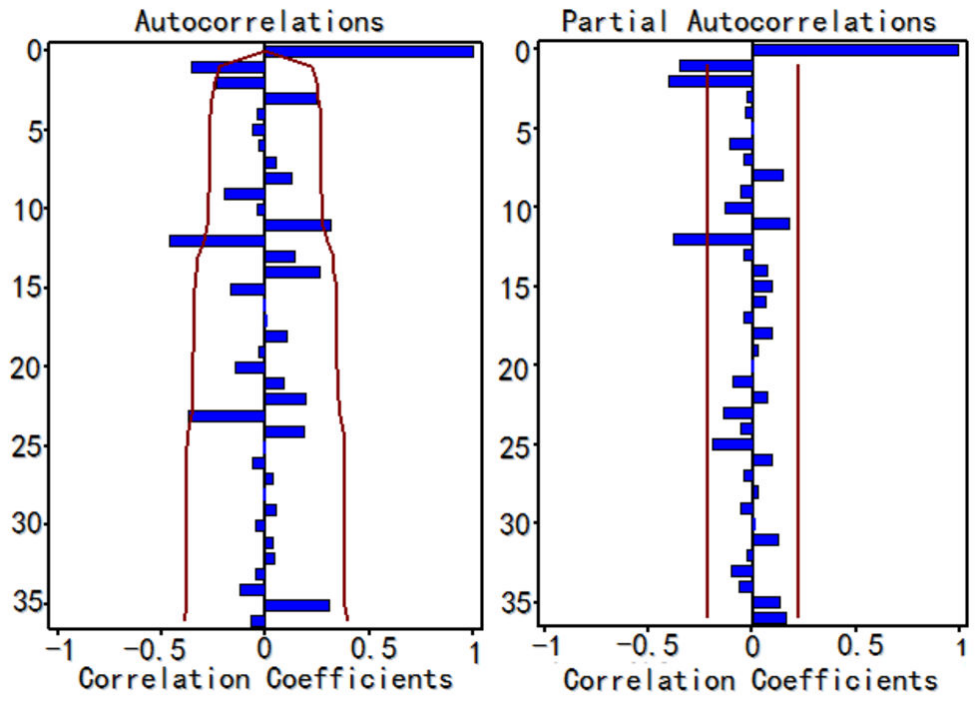
The ACF graph and PACF graph of the ARIMA (0,1,0) × (0,1,0)_12_ model. ACF=autocorrelation function, PACF=partial autocorrelation fuction. After taking a non-seasonal and seasonal difference, the TB report rate series shows dominant peaks in the autocorrelation functions lags 12, 24 and 36 months indicating a strong seasonal pattern in the report rate of TB in Hubei Province, China.

**Figure 3:**
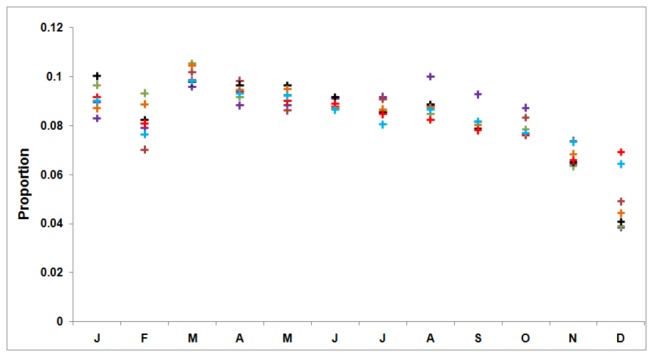
Proportion of tuberculosis cases by month of report. Month begins with January and is abbreviated by first letter. It shows that TB cases exhibit a peak in March and a trough in December across the entire study period.

Dominant peaks in the autocorrelation functions lags 12, 24 and 36 months indicates an obvious cyclical pattern (Figure 2). Figure 3 shows that monthly TB cases exhibit a peak in March and a trough in December across the entire study period. Of the 465,960 TB cases, 22.453% (95% CI: 21.432%, 23.369%) were in winter (December–February), 22.992% (95% CI: 22.407%, 23.605%) in autumn (September–November), 26.116% (95% CI: 25.431%, 26.869%) in summer (June–August) and 28.439% (95% CI: 27.935%, 28.938%) in spring (March–May). The proportion of cases was highest in spring (P < 0.001), second highest in summer (P < 0.001) and there was no significant difference between autumn and winter (P = 0.636). 

### Basic ARIMA model

**Figure 4:**
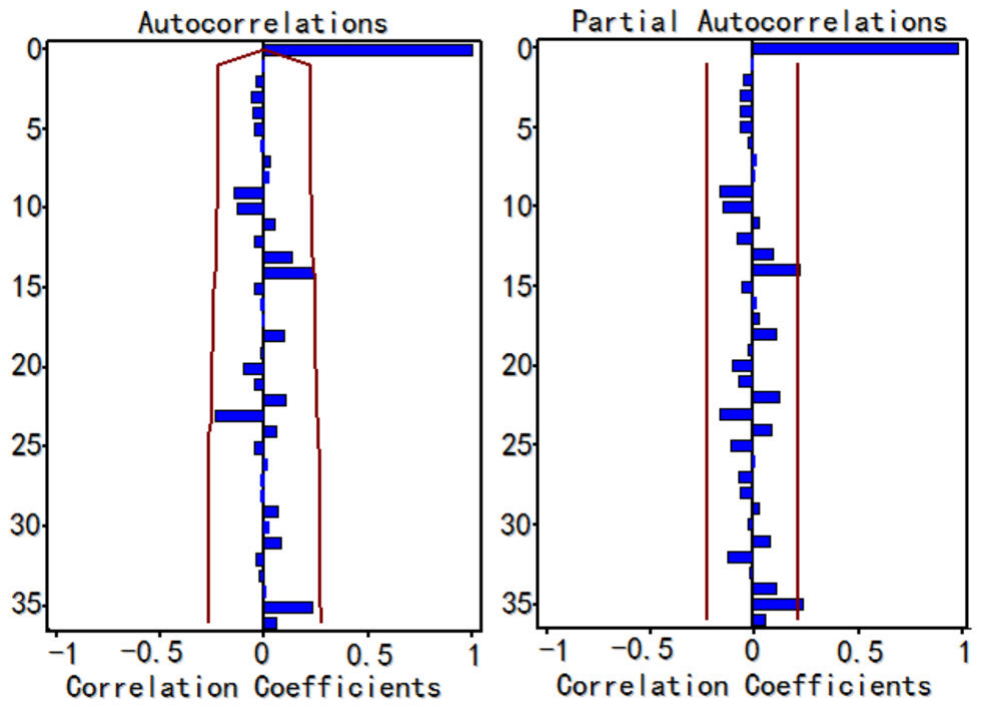
The ACF graph and PACF graph of the residuals for the ARIMA (2,1,0) × (0,1,1)_12_ model. ACF=autocorrelation function, PACF=partial autocorrelation fuction. As their correlation values are not outside the confidence intervals (CI) limits, the residuals error is considered to be white noise indicating that this model is appropriate for prediction.

Since the time series shows a declining trend and a cyclical pattern, we took a seasonal and non-seasonal difference equation to stabilize the series. After that, the series showed no trend and the result of unit root test was statistically significant (P < 0.01), which confirmed that there was no trend. Since the ACF and PACF graphs didn’t show an obvious pattern, a series of candidate models were tried. Results of the parameter test and residual tests helped to suggest alternative models. Among these potential models, some models were excluded as they could not pass the parameter or residual test. Three models were then taken into consideration: ARIMA (0, 1, 1) × (1, 1, 0)_12_, ARIMA (0, 1, 1) × (0, 1, 1)_12_ and ARIMA (2, 1, 0) × (0, 1, 1)_12_. It was found that the ARIMA (2, 1, 0) × (0, 1, 1)_12_ model fit the historical data best (R^2^=0.7840) with the MSE (0.6521) and SBC (-22.2328) smallest and AIC (-29.4894) second smallest in the plausible models (Table 1). Smaller AIC values suggest a better model and the SBC value considers the residual error, which is more universal. The parameter test results of the ARIMA (2, 1, 0) × (0, 1, 1)_12_ model are shown in the Table 2. Figure 4 displays the autocorrelation function paragraph and partial autocorrelation graph of the model residual series. As their correlation coefficients are not outside the confidence intervals (CI) limits, the residual is considered to be white noise. The result of the residual white noise test was not statistically significant (P > 0.1) which confirmed that there was no linear information left in the residual series. Thus, the ARIMA (2, 1, 0) × (0, 1, 1)_12_ model was selected to predict TB incidence in Hubei Province in the next 6 months.

**Table 1:** Comparison of three potential ARIMA models.

**Model**	**R^2^**	**MSE**	**SBC**	**AIC**
ARIMA (0, 1, 1) × (1, 1, 0)_12_	0.7810	0.6616	-25.4473	-30.2850
ARIMA (0, 1, 1) × (0, 1, 1)_12_	0.7790	0.6691	-24.5173	-29.3550
ARIMA (2, 1, 0) × (0, 1, 1)_12_	0.7840	0.6521	-22.2328	-29.4893

ARIMA=the autoregressive integrated moving average; R^2^ =coefficient of determination; MSE=mean square error; AIC=Akaike information criterion; SBC=Schwarz Bayesian information criterion.

**Table 2:** Estimate parameters of the ARIMA(2,1,0)×(0,1,1)_12_ model.

**Model parameter**	**Coefficient**	**Std. Error**	**t statistic**	**p-value**
Autoregressive, lag1	-0.4712	0.1054	-4.4717	0.0001
Autoregressive, lag12	-0.3409	0.1021	-3.3388	0.0013
Seasonal moving average, lag12	0.3910	0.1144	3.4187	0.0010

ARIMA=the autoregressive integrated moving average

### Hybrid GRNN-ARIMA model

**Figure 5:**
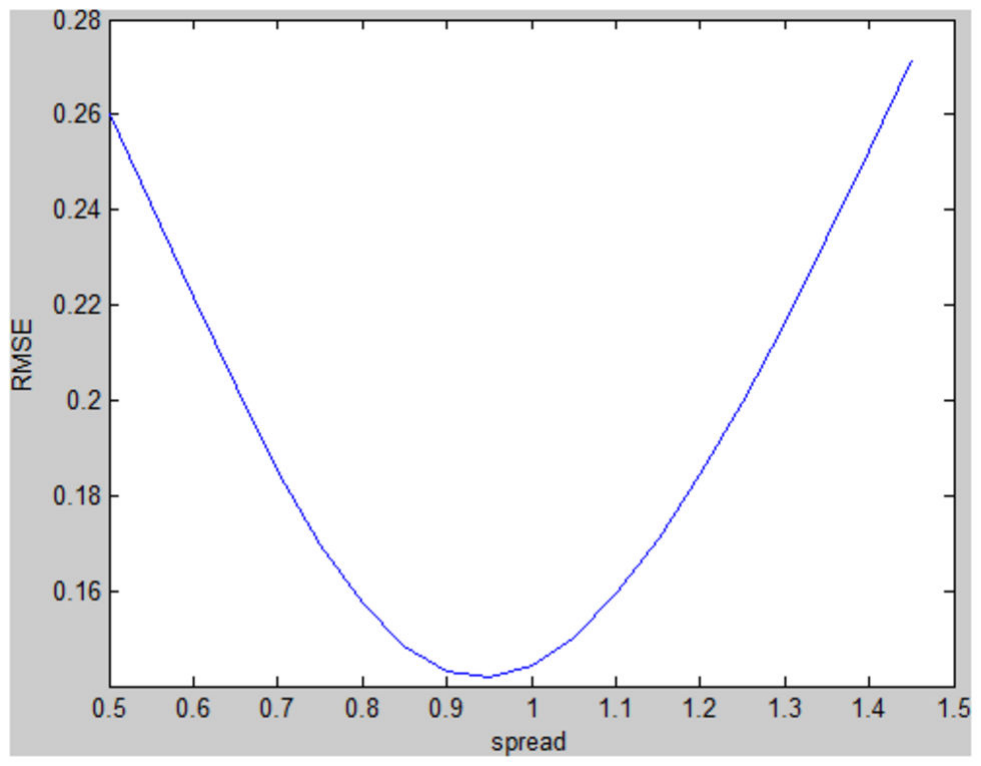
The selection of the spread of the GRNN-ARIMA model. ARIMA=the autoregressive integrated moving average; GRNN=the generalized regression neural network. The spread values between 0.5 and 1.5 with an interval of 0.05 were selected to find the minimum RMSE for the testing samples. When the spread was 0.95, the RMSE was lowest.

Because of the seasonal and non-seasonal differences, information on thirteen samples was lost. The fitted values of the ARIMA model and the actual values between February 2005 and Dec 2011 were used as the input and output of the GRNN, respectively. The observations of Jan 2007 and Jun 2009 were randomly selected as the testing samples to determine the spread. The spread values between 0.5 and 1.5 with an interval of 0.05 were selected to find the minimum root mean square error (RMSE) for the testing samples. When the spread was 0.95, the RMSE was lowest (Figure 5) and the RMSE increased continuously when the spread was lower than 0.5 or higher than 1.5. Therefore, the spread was determined as 0.95 and the GRNN was developed. Subsequently, the prediction values of the ARIMA model from Jan 2012 to Jun 2012 were used as the input of the constructed GRNN, and the output were the prediction values created by the hybrid model. 

**Figure 6:**
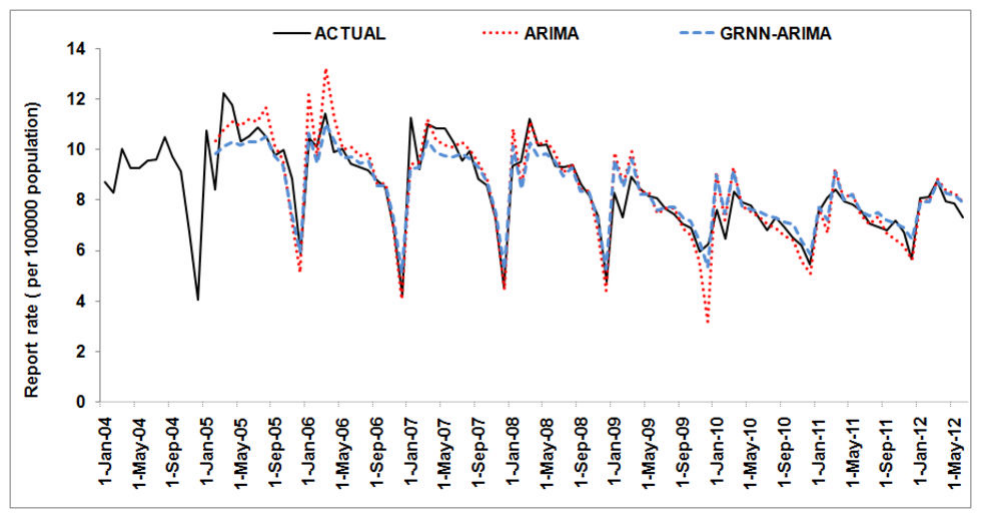
Two models’ fitting and prediction curves and the actual tuberculosis report rate series. ARIMA=the autoregressive integrated moving average; GRNN=the generalized regression neural network.

The prediction error of the hybrid model in fitting part and validation part was less than the ARIMA model, as MSE, MAE and MAPE showed (Table 3). Figure 6 depicts two models’ fitting and prediction error curves. Both models predicted that the TB report rate would have a slight increase in the first half of 2012. These data demonstrated that the hybrid model was more accurate and stable than the ARIMA model.

**Table 3:** Comparison of the fitting and prediction performance of the two models.

**Prediction**	**Fitting part**		**Validation part**	
**error**	**ARIMA**	**GRNN-ARIMA**	**ARIMA**	**GRNN-ARIMA**
MSE	0.6521	0.4467	0.1133	0.0958
MAE	0.5688	0.4966	0.2800	0.2555
MAPE	0.0677	0.0602	0.0361	0.0330

ARIMA=the autoregressive integrated moving average; GRNN=the generalized regression neural network; MSE=mean square error; MAE=mean absolute error; MAPE=mean absolute percentage error.

## Discussion

The sharp increase in the report rate of TB in 2005 and the different fluctuation pattern of report rate between 2004 and 2005 may be attributed to the implementation of the special report system for TB in that year, although the report rate was thought to have been reliable since 2004. Further improvement of the reporting system may act as an accident factor to the series, leading to a rise and change of TB case notifications. This may not be the real trend in TB incidence. Since there was no change in the TB reporting mechanism, the gradual decline may be attributable to the effect of intensive measures on TB including improved case notification, strengthened government commitment and leadership, increased TB budget, and improved public health system [16,20,21]. The slight increase in 2011, however, indicates that some specific measures should be taken to achieve the ultimate goal of eliminating TB in China. 

There is a striking seasonal variation in the series, with a precipitous decline in the report rate at year’s end, followed by an abrupt increase to an annual peak at the start of the next year. The seasonality of TB has been observed in many places such as Spain, United States, and Australia, etc [4,22–24]. Xinxu Li, et al [25] has evaluated the TB seasonality in China based on the national TB reported cases from 2005 to 2012. They found that the TB report rate was highest between March and May and lowest between January and February. Chi Chiu Leung, et al [26] found that TB cases in Hong Kong between 1991 and 2002 showed a trough in either January or February and a peak between May and August. In this study, the TB report rate in Hubei Province was also highest between March and May but lowest between October and December. This difference may be attributed to the wide disparities between different areas in China where the lifestyle and climate factors are always different. These differences also indicate that more studies need to be conducted in China to explore the TB seasonality in various places there. The seasonality of TB was deemed to be associated with factors such as indoor exposure, sunlight exposure, vitamin D levels, or temperature, etc [25,27,28]. It is probably a genuine biological phenomenon. However, this pattern also suggests that the surveillance system may have delays. For example, many cases presenting at year’s end may be not promptly entered into the system because of the Spring Festival. People without obvious or intolerable symptoms may not go for check ups at that busy time [29]. The population mobility during this period will make surveillance more difficult. In addition, it is easier for people to get other respiratory infections in winter which have symptoms similar to TB, resulting in the ignorance of diagnosis of TB [23]. These patients will be held over until the start of the next year. Thus, the apparently increasing report rate at the beginning of the next year may be exaggerated by the backlog of cases from the previous reporting period. Further study needs to be conducted to clarify the seasonality in Hubei Province and then provide suggestions for influencing factors of TB epidemic and relevant measures. Also, the government should determine whether the decline in TB notifications at the end of the year is caused by delayed diagnosis or reporting. This process is necessary because delayed reporting of TB will not only jeopardize the accuracy and reliability of the surveillance system and then influence the quality of an early warning system, but will also threaten the healthy people and lead to a broader transmission of TB. If the delay does exist, the government should invest more resources in surveillance at that time, such as conducting more extensive publicity and setting more convenient monitoring points to encourage patients to undergo examinations. Meanwhile, surveillance data at the start of the next year should be adjusted by distinguishing the delayed patients from the new cases. If the delay does not exist, the government can put more resources into the treatment and control of TB in the periods with highest recorded incidence. The surveillance data, therefore, can be used with confidence to direct programmatic interventions and allocate public health resources.

The prediction model can forecast expected numbers of cases for a given number of future time intervals to provide quantitative directions for the allocation of health resources. For example, the expected number of cases from Jan 2012 to Jun 2012 created by the ARIMA model and hybrid model are 28,446 and 28,275. The actual number is 27,753. As the hybrid model has better prediction accuracy, as shown in this study, it is reasonable to use this model as a decision-supportive tool when making health resource budgets. According to the principle of the GRNN, adding more surveillance data will improve the prediction accuracy as more information about the underlying relation between the history data and the future values will be collected. Also, the hybrid model will show a larger advantage when the high-precision ARIMA model is not found.

This model can be applied to forecast the monthly trend of TB incidence, which will contribute to planning public health interventions. For example, based on the prediction results, the government can invest more health resources during high-risk periods and decrease it during low-risk periods to improve the cost-effectiveness of interventions and scheduling of resources [30]. It can also be used to evaluate the effectiveness of public health interventions under varying assumptions by comparing actual TB incidence with expected incidence.

Combining linear models with neural networks has achieved good results in predicting time series in economics and other areas. Theoretical and practical results have both proved that a hybrid model may improve forecasting performance. However, few hybrid prediction models have been used to predict TB incidence in China. Shiyi Cao, et al [31] constructed a SARIMA-GRNN model with the data of national TB reported cases from Jan 2005 to Dec 2011. Because the ability of reporting TB cases is different in various places and the magnitude and pattern of TB vary with regions in China. Thus, a prediction model based on the provincial data, especially in a high-burden province like Hubei, may have a more directive and practical value. Also, they developed the prediction model with the number of reported TB cases but not the TB report rate. Without considering the change of population, the number of reported TB cases may not be able to reflect the real TB incidence well. The large number of reported TB cases will also make the construction of the hybrid model harder because the complexity of neural network was added. Compared with the former study, we determined the optimized ARIMA model after examining a series of plausible models to ensure its quality. When constructing the hybrid GRNN model, we determined the spread using the method proposed by Specht. Two samples were randomly selected to ensure the optimized spread. The spread lower or higher than the set range were also examined for further confirmation. These works may help us to determine the best model. We both found that a hybrid model had a better forecasting accuracy than the ARIMA model alone, indicating that it may be applied in various places. We also compared the fitting performance of the two models in our research. The hybrid model was also superior. 

It should be noted, however, that the combination process of different models is not arbitrary. When selecting the constituent models, not only the model’s prediction accuracy but also the fluctuation characteristics of raw data should be considered. The constituent models are better to have different principles and relatively high prediction accuracy to heighten their merits [12,32]. In the present study, the hybrid model was developed based on the ARIMA model, thus selecting the best ARIMA model is important. The ARIMA model requires at least 50 observations to fit and recognize the potential relationship in historical data [6]. 

There are some limitations in this study. First, the performance of the hybrid model needs more validation in the future because the model was constructed based on data from one province in China. Second, this model did not include some variables such as climate factors which may offer more information. Last, as the ARIMA based model is a short-term prediction model, frequent updating by adding new data is necessary to maintain the quality and stability of the hybrid model. Further studies were expected to explore hybrid models based on other constituent models. As the transmission conditions of TB and health polices are not invariant, prediction models which can consider the influences of accident factors on TB are also needed.

## Conclusions

We developed a hybrid GRNN-ARIMA prediction model for the incidence of TB. This model is superior to the traditional ARIMA model alone and could improve public health responses and resource allocation in China.
